# The Use of Operational Excellence Principles in a University Hospital

**DOI:** 10.3389/fmed.2017.00107

**Published:** 2017-07-13

**Authors:** Eric R. Edelman, Ankie E. W. Hamaekers, Wolfgang F. Buhre, Godefridus G. van Merode

**Affiliations:** ^1^Faculty of Health, Medicine and Life Sciences, Department of Health Services Research, CAPHRI School for Public Health and Primary Care, Maastricht University, Maastricht, Netherlands; ^2^Department of Anesthesiology, Maastricht University Medical Center+, Maastricht, Netherlands; ^3^Executive Board, Maastricht University Medical Center+, Maastricht, Netherlands

**Keywords:** operational excellence, university hospital, perioperative, lean six sigma, Toyota Production System, target costing, process optimization

## Abstract

The introduction of Operational Excellence in the Maastricht University Medical Center (MUMC+) has been the first of its kind and scale for a university hospital. The policy makers of the MUMC+ have combined different elements from various other business, management, and healthcare philosophies and frameworks into a unique mix. This paper summarizes the journey of developing this system and its most important aspects. Special attention is paid to the role of the operating rooms and the improvements that have taken place there, because of their central role in the working of the hospital. The MUMC+ is the leading tertiary healthcare center for the South-East region of The Netherlands and beyond. Regional, national, and international developments encouraged the MUMC+ to start significantly reorganizing its care processes from 2009 onward. First experiments with Lean Six Sigma and Business Modeling were combined with lessons learned from other centers around the world to form the MUMC+’s own type of Operational Excellence. At the time of writing, many improvement projects of different types have been successfully completed. Every single department in the hospital now uses Operational Excellence and design thinking in general as a method to develop new models of care. An evaluation in 2014 revealed several opportunities for improvement. A large number of projects were in progress, but 75% of all projects had not been completed, despite the first projects being initiated back in 2012. This led to a number of policy changes, mainly focusing on more intensive monitoring of projects and trying to do more improvement projects directly under the responsibility of the line manager. Focusing on patient value, continuous improvement, and the reduction of waste have proven to be very fitting principles for healthcare in general and specifically for application in a university hospital. Approaching improvement at a systems level while directly involving the people on the work floor in observing opportunities for improvement and realizing these has shown itself to be essential.

## Introduction

The introduction of Operational Excellence in the MUMC+ has been the first of its kind and scale for a university hospital. The policy makers of the MUMC+ have combined different elements from various other business, management, and healthcare philosophies and frameworks into a unique mix. Internally, this has become known as the Maastricht Operations System (MOS). This paper strives to summarize the journey of developing the MOS and the most important aspects of it, so that others may use it as case study. Special attention is paid to the role of the operating rooms (ORs) and the improvements that have taken place there, because of their central role in the working of the hospital. We hope that hospital administrators, healthcare managers at all organizational levels, medical professionals, and anybody else interested in high-efficiency, bottom-up healthcare organizations may benefit from our experience.

To understand the motives behind the massive undertaking of redesigning the MUMC+’s mode of operation, we must first study the characteristics of the hospital and the events in the years leading up to actual deployment of Operational Excellence.

## The Hospital in a Nutshell

The MUMC+ can trace its history back to the 10th century infirmary founded in Maastricht by the monks of Saint Servatius. Many centuries later, it has transformed into the leading tertiary healthcare center for the South-East region of The Netherlands and beyond. By integrating patient care, science and research, and education, the MUMC+ strives to provide the best possible care and health improvement possible. In the areas of cardiovascular disease, respiratory disease, oncology, and neuro-intervention, the collaboration between the University of Maastricht and the hospital is especially tight, resulting in work being done at any scale from molecule to population. Other clinical focus areas include mobility, ophthalmology and heredity, reproduction, and early development.

The hospital has 715 beds and 26 ORs, broken down into 15 in-patient ORs, 4 day case surgery ORs, 3 ORs for patients undergoing local anesthesia, and 4 ORs for ophthalmology under local, regional, or topical anesthesia. In 2015, 27,537 patients were admitted who stayed for an average of 7.1 days. 21,801 patients were surgically treated as outpatients, while the outpatient clinics were visited a total number of 435,168 times. 27,774 emergency patients were treated.

That same year, the university Faculty of Health, Medicine, and Life Sciences had 4,856 students enrolled of which 1,713 were new. Ca. 2,300 academic papers were published.

The MUMC+ is a major employer for the region, providing work for 7,239 people (equivalent to 5,920 full-time positions). Furthermore, 57 other companies have found a home on campus.

## Drivers of Change

In the years 2005–2010, a growing sense of urgency developed within the MUMC+ leadership that the hospital needed to keep up with multiple developments. Regional, national, and international competition was increasing. Collaboration with other centers was desirable and required a stable economical and organizational status to attract other parties. Employees too would need to be encouraged, as the Dutch healthcare sector is expected to need between 700,000 and 1.4 million additional people for its workforce by 2040 (1).

In July 2011, the pace of change accelerated when the Dutch Ministry of Public Health, Welfare and Sport, major healthcare provider associations, and health insurance companies agreed on the so-called “Governing Framework Agreement for Medical Specialized Care.” The main objective was to control national healthcare expenditures and improve hospital quality by limiting the growth of expenses for hospital care to 2.5% per year for 2012–2015, despite an increasing demand for care. In addition, insurance companies announced that they would increase their efforts to selectively purchase care by evaluating price, quality, efficiency, and appropriate usage. The healthcare providers and insurance companies would work together to spread out and concentrate hospital-based care to offer their services as close to patients as possible. Hospitals would dismantle superfluous capacity to increase efficiency.

The MUMC+’s strategy developed in the years before anticipated many of these developments. It emphasized the importance of focusing on “the human as a whole,” instead of only the diagnosis and treatment of diseases and disabilities. This implies paying attention to the environment and disposition of each patient. Strategic ambitions set for 2020 included increasing preventive efforts and helping to improve the living environment and lifestyle of people throughout the region. (Even though reimbursement of such efforts has still not yet fully materialized.) The hospital’s social focus is to keep prevention and care affordable for all. These guiding principles put the MUMC+ in a strong position to face the changing environment it operated in.

In addition, the hospital enjoyed excellent infrastructure (with ambitious plans for improvement and expansion), a healthy financial position and the status of product leader, with the hospital and university both scoring well in national benchmarks.

But product leadership alone is not enough to survive in the long term. To face all current and future challenges and fulfill its ambitions, much improvement was still needed. In general, both medical staff and management felt there was insufficient focus on the patients and their individual needs. In industry terms, the organization was running mainly as a “push” system (supply driven), instead of a “pull” system (acknowledging actual demands) (2, 3).

In the years before, the MUMC+ lacked an encompassing operations strategy. Like most hospitals, the focus had always been on capacity optimization instead of stream optimization and process management in general. Unsurprisingly, this meant the MUMC+ suffered from the same issues so many other hospitals faced. Care processes were insufficiently standardized, which caused errors and various forms of waste. This also made planning complex and difficult, leading to the frustrating situation of unused capacity, on the one hand, and people and processes waiting for each other, on the other hand. Using the OR complex and its perioperative processes as an example illustrates the difficulties faced. Patients were put on waiting lists before being admitted. Once at the hospital, they waited on the various steps needed for their diagnosis, preoperative preparation, and surgical treatment. Meanwhile, doctors and nurses waited for patients, for each other and for infrastructure such as beds and machines to become available (3, 4). These healthcare professionals were making things work by putting time and effort into activities that should not be required of them.

To continue on its strategic course, the MUMC+ needed to significantly reorganize its care processes. The way care was being provided was driving up costs and stopping the improvements desired in quality and other outcome measures. Throughout different layers of the organization, the realization set in that optimizing the MUMC+’s business processes was a basic condition to realize its ambitions. Following the definition of business processes in healthcare as defined by Vanwersch et al., this included “the steps, from intake until aftercare, that are performed for a patient care request.” Examples include not only steps related to diagnosis and treatment but also supporting steps such as making appointments (5).

These ideas needed to be translated into defined, realistic, and evaluable goals, which in turn would be linked to the leadership and organizational culture, reducing unclarity and inconsistency in control. The attending physicians were expected to play a key role in this, by taking ownership of processes and taking the lead in improving them.

In short, what was needed was a comprehensive system that would give the right people the right tools to improve and keep improving the hospital’s processes.

## First Experiments

At the end of 2009, the departments of Orthopedics and Dermatology had already independently started rethinking their work methods. They recognized that their systems (scheduling and processes) were relatively “unstable,” meaning they produced varying results due to unidentified sources of variation. In addition, previous improvement efforts had resulted in isolated successes, without sustained overall improvement and cohesion between results.

These initiatives were the perfect testing ground for developing an improvement strategy that could later be rolled out across the entire organization. Orthopedics and Dermatology started piloting the use of tools from Lean Six Sigma^1^ and Business Modeling in 2010. Although the hospital’s top management also received training on these topics, they realized that medical professionals are extensively educated people who, through their jobs, are used to thinking autonomously and taking responsibility for their actions. Their collective brainpower was explicitly made use of by asking them to “think like designers.” Medical staff was given the training and tools to design their own business model.

Not everybody embraced these new tasks and some even left the organization. The rest, however, were convinced and organized themselves into a management committee for each of the two departments. These committees then appointed teams which consisted of all relevant personnel, from attending physicians and nurses to paramedical staff and receptionists. Often, these people had never sat together in this formation before. The resulting knowledge sharing was highly inspiring. The board of directors facilitated this process, but only imposed one thing: that patients would be included after the second joint session, so that the teams would receive feedback as quickly as possible from the patient’s perspective. It would also drive the teams into operational mode. The strategy would not be allowed to devolve into a theoretical exercise.

Both departments independently designed and implemented new care models with corresponding financial models, customized for their patient populations. This was perceived as a major advantage. In a university hospital, an overall strategy may guide the organization in a general direction, but the differences per business unit regarding customers/patients, technologies, and processes are so great that only a bottom-up approach provides the units the opportunity to finetune their way of working to their specific situation.

The results were encouraging. Among other things, the Lean way of thinking uncovered underutilized space and demonstrated that for the doctors the ratio of time spent on patient bound activities versus non-patient bound activities could be improved. These issues were familiar to staff working in various other departments, including the OR complex, and in 2011 the decision was made to perform an organization-wide rollout of selected aspects of Lean Six Sigma to form a new organizational development strategy. An immediate goal was to prevent any further downsizing, as had been necessary in the previous year, when the organization size was reduced by 260 full-time equivalents.

## Learning from Others

In the spring of 2012, it was becoming clear that many medical doctors, who were supposed to play a leading role in the improvement projects throughout the hospital, either were not able to combine the management of processes with their daily tasks or simply were not sufficiently interested in assuming this extra role. Many of them did not trust the organizational reconstructions going on. Management needed inspiring examples to demonstrate the benefits of process-oriented thinking and what a (financially) healthy care model looks like.

To this end, senior management expanded the existing learning relationship with Aravind Eye Care System in Madurai, India. This small hospital started with 4 doctors and 11 beds and has since grown to be one of the leading medical providers in India, with specialized hospitals all over the country reaching 2.67 million people for ophthalmological care. The reality of scarcity has driven them to optimize their processes to the extreme. The cost of performing cataract surgery at one of their centers can be as low as $42, without compromising quality (7). Their mission to provide care to everybody in need, regardless of ability to pay and social status, dictates the use of “target costing,” i.e., determining an affordable price beforehand and adjusting processes to that (See the section “The MOS” for more information on the role of target costing at the MUMC+).

Aravind and comparable examples displayed the power of process optimization (8). Further visits were made to Narayana Hrudayalaya (a leading hospital chain in India, currently named Narayana Health), Boeing, Medtronic, Virginia Mason Medical Center, and the Seattle Children’s Hospital to learn more about their work methods. It also raised an important point on wording, which was especially important to positively influence and enlighten the medical staff. Talking about a hospital in terms of a “factory” had traditionally been frowned upon by medical professionals, invoking in them feelings of rushed production line work without room for personalized care. It now became clear that production line models and similar concepts were not incompatible with providing quality care and often even increase quality, volume, or both. Important concepts and methods involving customers, quality, and efficiency can be used outside of production line environments.

Using these examples, the MUMC+ management was able to convert the majority of the medical staff from skeptics to some of the most enthusiastic propagators of the new way of thinking. It was becoming clear what the MUMC+ wanted to be and how it was supposed to generate revenue. In addition, the practical and scientific approach of hypothesis-driven experiments leading to improvement appealed to doctors.

Before moving ahead, the board of directors realized at this stage that sticking purely to Lean Six Sigma tools would not be sufficient to realize their vision. Lean projects are generally aimed at improving things from the perspective of the organization and not from that of the customer. Also, many Lean projects limit themselves to increasing the efficiency of existing processes, instead of rethinking the whole system. What senior management had in mind was broader than that.

Other incompatibilities surfaced by doing as well. The essence of Lean is cost reduction, meaning outcome measures are the actual cost reduction achieved and the return on investment of projects. Line managers delegate the execution of projects to experts, instead of being directly involved. Senior management only monitors progress, using regular reports, key performance indicators, financial data, and checklists. The results often get stuck at short-term, local cost reductions, without maturing of the organization as a whole by improvement of the coordination between elements (9). Perhaps the most important factor missing was a system and culture in which the patient is truly at the center.

These insights led to elements of the Toyota Production System^2^ being included in the final framework applied to the organization of the MUMC+. This framework is known as “Operational Excellence” and in November 2012 the board of directors unanimously agreed to making it one of the three pillars of the hospital’s strategy, besides the centers of clinical focus (as mentioned in the section “The Hospital in a Nutshell”) and the forming of a true university medical center from the University of Maastricht and the hospital. This way, the defining goal remained creating value for the patient by providing quality care for an affordable price.

In its execution, the plan called for widespread training of employees to the levels of yellow belt (an introduction to Operational Excellence) or green belt (leading improvement projects). Having many people involved early on reduced the risk of grinding to a halt due to a lack of action. It also helped spread the new culture throughout all layers of the organization. Skepticism and criticism were avoided by quickly bringing everybody into contact with actual improvement efforts instead of introducing these through long discussions. By the end of the year, dozens of projects had been started by employees throughout the hospital.

To better support the growing number of projects, all first and second echelon management were expected to participate in seven-day executive belt training by the second half of 2013. All participants were trained in various concepts such as the cycle of define, measure, analyze, improve, and control. Those training to be lean managers received additional training in using structured analysis and improvement plans and improvement boards.

By 2014, the training had reached a stable situation. 1,700 employees had been trained as yellow belts, 80 as Lean practitioners/green belts. 140 people were being trained to be Lean managers. Five (master) black belts had formed a program office, leading the rest and providing support during projects where required. (See the section “The Maastricht Operations Systems” for more information on the role of the program office in the MUMC+.) Medical staff were involved in 26 Operational Excellence projects focused on patient care. Entirely new concepts were being developed for the wards and the outpatient clinics based on Operational Excellence.

## Obstacles

Superficially, things seemed to be going very smoothly. Many employees had completed some level of training, a large number of projects had been started, and the new culture of process orientated improvement seemed to be spreading through the organization. However, a deeper and frank evaluation in September 2014 brought a number of warning signs to the steering committee:

– Nearly all Lean practitioners/green belts started at least one project, usually as part of their training, but more than half never started a second project.
– Despite the first projects being initiated as far back as 2012, 75% of all projects had not been completed. The majority of these were small-scale initiatives that had been started with a very limited scope, meaning the project structure was often not strictly necessary. It was specifically this type of project that was often not completed.
– Over 40% of the Lean practitioners had no mentor or did not make use of their mentor.
– Despite having received the training, hardly any of the Lean practitioners/green belts had been certified.
– Part of the projects was being executed based on poor charts and/or without any resulting benefit for the patient.

An evaluating committee of the program office summarized these issues as the presence of “a very real risk of insufficient uniformity and incorrect application of instruments.”

An interesting cultural discussion developed, in which it became clear that employee trust in management based only on knowledge and experience, was not sufficient. Despite the enthusiasm for change in 2012, not everybody seemed wholly convinced of the new system. Other factors that hindered execution were the traditionally bureaucratic processes of the hospital and inexperience of staff in dealing with implementation.

A number of policy changes were made to improve the situation. Monitoring was intensified. Project charters would from now on need to be approved in the Define phase by a champion and the mentor (senior staff directly involved in coaching and supporting the project team). A structured analysis and improvement plan would be approved at each follow-up phase by the mentor and at delivery by the champion and the mentor again. This way of working offered the added advantage that all project documentation would be complete and made available to all interested employees via the internal network.

In conclusion, the first 18 months of Operational Excellence at the MUMC+ showed that the employees were very capable of designing and implementing small-scale projects themselves, but that they occasionally needed some extra support for larger scale projects. This resulted in a more top-down management style than originally intended to keep a lookout for blind spots and, if necessary, provide subtle course corrections.

## Making Progress

During the introduction of Operational Excellence in the MUMC+, a lot was learned through pioneering work and this process continues today. At the time of writing, many improvement projects, ranging from hospital real estate design to the throughput time of an update to a patient information leaflet, have been successfully completed. Various examples are summarized in Table 1.

**Table 1:** Various examples of improvement projects throughout the hospital.

Organizational level	Examples of completed improvement projects
Hospital real estate	New designs for the wards, the operating rooms (ORs) complex, the outpatient clinics, and the Accident and Emergency Department
“Result Responsible Units” (broad organizational units in the hospital, such as Surgical Medicine, Imaging and Laboratory etc.)	Redesign of the Oncology center and the Mobility Center Improvement of the collection of management and quality-related data Streamlining the procedures for new employees
Operating room complex	Reduction of the number of blood products ordered by ca. 50% Elimination of waiting time for surgical treatment of hip fractures by implementing a 2 h trauma slot in the OR planning per day Reduction of late starts of operations due to missing equipment (from 1% of operations to 0.3%)
Medical specialties	Simplification of the procedures surrounding reimbursement by the healthcare insurance companies
Local	Reduction of the average duration of stay for patients in the wards (from 9.0 to 7.2 days) Reduction of waiting times for the outpatient clinics (for example Orthopedics from 36 to 14–21 days) Reduction of waiting times in the outpatient clinics (reduction of throughput time of patients with hematuria from referral to diagnosis by 56%) Improved information on absenteeism
Supporting processes	Reduction of the throughput time of an update to a patient information leaflet (from 73 to 45 days)

The OR complex is an especially interesting area when striving for improvement. Surgery is a major cost driver (12) and is dependent on many other processes in the hospital. In addition, roughly a third of patients at the MUMC+ are surgically treated, making it a major influence on patient outcomes and satisfaction.

A good example of how surgery-related workflows are improved is the pediatric ear, nose, and throat (ENT) program. Independent observers were present during two standard programs of pediatric ENT cases. The surgical team was especially selected from experienced volunteers, who were willing to look critically at processes they may have been part of for years. The observers made detailed notes on all aspects of how the work was being performed. In some cases, staff were asked additional questions to clarify their activities and why these were being performed in a specific manner. It is important to note that the recorded observations were not necessarily all opportunities for improvement. The observers did not have the expertise to judge how the workflows in this specific case should be optimized. (Although they made suggestions to stimulate the staff involved to think about alternatives.) Instead, all observations were discussed afterward with the staff involved.

The observations were grouped into several themes: communication, standard operating procedures, last-minute/*ad hoc* activities, inefficiency, materials, and patient experience. Every theme produced various points that are currently being improved. Examples include synchronization of working and break times for all involved staff; a morning briefing with the entire surgical team of the day; designated staff members for specific tasks, such as requesting the next patient; and standardized anesthetic procedures that are also known to the surgeons, so that various steps in the operation may be timed correctly. Other points are unfortunately not possible to change yet, either for regulatory or organizational reasons. Examples of these include preparing anesthetic medication for the next patient before he/she has arrived, reducing variability in anesthetic emergence duration, bringing up staff skill levels to the same level, and updating information technology infrastructure.

An example of an important overall theme driving projects undertaken in the ORs is improving the timely start of operations. The first aspect to be targeted was the availability of mobile equipment. Roughly 1% of operations were starting too late (with a median delay of 17.5 min) due to missing equipment. Staff interviews revealed several reasons: the OR order in the computer system either did not describe the needed equipment in sufficient detail or the information was spread over too many entry fields, staff responsibilities were unclear regarding the collecting and preparing of mobile equipment and there was a lack of standardized storage space for the equipment. In response, the computer system was improved, a list was compiled containing every piece of equipment needed for each type of operation and standardized storage space was assigned and made recognizable by labeling the equipment and the spaces with codes. A short while later, maps of the OR complex were created, displaying the various storage spaces and the equipment intended to be kept there. The combination of these interventions was a success: the number of delayed operations due to missing equipment was reduced by 68%.

Interestingly, other attempts at improving the rate of late surgical starts have been less successful. For example, new working procedures were drafted for the OR staff working in the evenings and at night. These were intended to standardize the way the OR was left behind after the last operation of the night, so that everything would be ready for the first operation of the next day. In this case, although staff appreciated the improved clarity of a standardized way of working, there was no reduction in the number of late starts of the first operation of each day.

A project that does not achieve its intended outcome is not a problem. Operational Excellence is also about trying out many different things, testing them, and concluding which should be continued and which should not. Sometimes, a project by itself might be promising but the dependency on other processes does not allow it to come to fruition. This is often the case in a unit as central to the hospital as the OR complex. When an attempt was made to improve the planning and sequencing of operations, it turned out that not all the necessary information was available yet, especially concerning inventory and logistics. Information was often missing or incomplete and staff complained that manual entry was cumbersome. Because this situation will change with the introduction of Material Management in the hospital, it was decided to revisit the improvement of the OR schedule at a later date.

## The MUMC+ Today

Every single department in the hospital now uses Operational Excellence and design thinking in general as a method to develop new models of care. These models include not only health restoration but also preservation and improvement. In response, the board of directors has assumed a new role. They no longer dictate a strategy but are responsible for connecting all the different departments. This also includes resource sharing, such as imaging and laboratory technology, but also expertise.

The policy changes made after the evaluation of September 2014 have been continued. The relevant lesson learned is that people are inclined to start many pilots and projects, which is commendable. Sometimes too much is started though and not enough finished. The timing of new initiatives needs to consider current running projects and, in some cases, guidance is needed for completion and implementation.

Employees are taking full advantage of the opportunity to improve or completely replace work processes in order to create more value. This has been highly successful in many places but not in every case. Some projects were successful locally but have failed to achieve high leverage elsewhere. The link to the hospital’s overall strategy and values is still growing.

Most importantly though, the redesign of care processes is starting to have its effect on management processes as well. Traditionally in healthcare, these are far removed from each other, but with care units designing their own business models, the worlds of patient care, on the one hand, and revenue and budgets, on the other hand, are increasingly integrated. Because the people who really understand healthcare and successful business models are now in the driving seat, it will be very exciting to see where they will take the hospital in the future.

Table 2 summarizes the vision for the MUMC+ regarding Operational Excellence. The patient replaces the care provider at the center of the system. Waiting is bad, errors should be minimized through system improvement, responsibilities need to be clear, new resources are not an answer, waste should be reduced instead of costs, real-time control instead of afterward, and direct involvement of management. Most important of all might be the last item: there is no time to lose.

**Table 2:** A summary of the vision for the MUMC+ regarding operational excellence.

Old situation	Ideal situation
Centered around healthcare provider	Centered around patient
Waiting is fine	Waiting is bad
Errors are unavoidable	Errors minimized through system improvement
Diffusion of responsibility	Clear responsibilities
New resources as solution	No new resources
Reduction of costs	Reduction of waste
Retrospective control	Real-time control
Management at a distance	Management directly involved
We have time	There is no time to lose

## The MOS

Although the policy makers of the MUMC+ would not go so far as to claim they have designed their own production system, they have combined different elements from various other business, management, and healthcare philosophies and frameworks into a unique mix. Internally, this has become known as the MOS. To conclude this paper, we will now summarize the most important aspects of this system.

### Leading Principles

The leading overall principles are that the realization of value for the customer (usually the patient, but this could also be the patient’s family and friends, MUMC+ colleagues, or even external parties such as insurance companies) is top priority in everything, that only activities that contribute to realizing that value should be pursued, that the complete process leading to this value should be optimized, and that all of this should be continuously improved. Improvement should not focus on any one area but on total optimization of quality, costs, throughput times, customer satisfaction, safety, and employee satisfaction. New measures for success are needed, including throughput times, the ratio of value adding activities to non-value adding activities, the number of transfers of information, distances walked, productivity per day (per doctor, employee, operating room), number of complications, etc.

A careful balance must be found between simplification and standardization, on the one hand, to efficiently create specific value while, on the other hand, still remaining flexible enough to produce sufficient product variety to cater to different target populations.

### Reducing Waste

Focusing on creating value automatically implies reducing waste. The following forms of waste are focused on:

– Inventory (documents, materials, machinery)
– Overproduction (space, over-prescription)
– Correction (apologizing for waiting, extra visits)
– Transport (samples, documents, materials)
– Processing (invoicing, dictating)
– Waiting (in waiting rooms, on the wards)
– Movement (searching, on the wards, etc.)
– Waste of talent (menial tasks, micromanaging, etc.).

### Synchronizing Streams, Push, and Pull Systems

Reducing waste is often a result of carefully synchronizing streams. The MUMC+ identifies the following types of streams of patients, patient materials, personnel, medication, materials, visitors, and information. The end goal is to have all departments synchronize their streams to each other, creating one tempo that the whole organization works by. Specific capacity is then adjusted to match the desired overall tempo. Any process should not work any slower than this tempo but also not faster.

The information stream is of special importance in a hospital, because so many operational aspects depend on it. Two complementary strategies may be combined to improve information processing: the level of required information processing may be reduced and the organizational capacity to process information may be increased. Once the required balance has been achieved, the hospital can start organizing activities as “pull” systems instead of the traditional “push” systems. Hopp and Spearman define these characteristics as follows:

A push system schedules the release of work based on demand, while a pull system authorizes the release of work based on system status. (2)

Examples of push are admission planning, OR capacity planning, the fixed allocation of beds, an inventory of drugs or other materials, and staff bound to specific departments. Examples of pull are patient scheduling instead of capacity allocation, flexible OR planning, and hospitals with minimal inventories.

### Leadership

The choice for Operational Excellence as a framework for the MUMC+ was not made lightly, nor was it without risk. From the beginning, it was understood that its success largely depended on the way the leadership would be able to combine a top-down introduction with an open field for bottom-up filling in and execution. The realization of Operational Excellence is, therefore, directly controlled by the board of directors, led by the chairman of the board, who is ultimately responsible.

Ideas propagate down by way of the heads of the “Result Responsible Units” (RRU; broad organizational units in the hospital, such as Surgical Medicine, Imaging and Laboratory, and so on), staff directorates, and staff services. The line managers are responsible for taking the initiative for improvement and executing their plans. Support is provided through the central and decentral program organizations.

The central organization is led by the board of directors and the Operational Excellence program directors. The central organization can be seen as a platform which further includes all first and second echelon management and representatives of the Employees Council (representing all employees), the Staff Assembly (representing the medical staff), and the Nurses Advisory Board (representing all nurses). Also included is the program office, consisting of five (master) black belts. These are generally tasked with supporting complex and/or RRU broad projects; coaching and mentoring current Lean practitioners and green belts; training new Lean practitioners/green belts and Lean managers; and coordinating and running the program office. It is important not to waste talent by making inefficient or ineffective use of the black belts.

The decentral organization consists of the Lean practitioners/green belts initiating and executing the decentralized projects.

MUMC+ leaders are expected to be culture bearers of Operational Excellence. They must be completely committed to the underlying philosophy. The hospital provides them with the necessary training in applying Lean management, but after that they must drive themselves to become experts in change and become active in executing projects. They are expected to “walk the gemba,” i.e., visit the work floor and observe the work methods and processes there for themselves. There is no substitute for direct supervision. They should coach their teams in finding improvements each and every day, without determining the solution themselves. Any change should be tested as experiment first and frequent experimentation is encouraged. Focus should be on the total process, not on individual elements.

## Conclusion

Rolling out Operational Excellence at the MUMC+ has fundamentally changed the way people work, think, and interact within the hospital. It has produced impressive results so far and there is more to come. The organization as a whole is continuously learning and is confidently on its way to the next phases of Operational Excellence.

Focusing on patient value, continuous improvement and the reduction of waste have proven to be very fitting principles for healthcare in general and specifically for application in a university hospital. Approaching improvement at a systems level while directly involving the people on the work floor in observing opportunities for improvement and realizing these has shown itself to be essential.

The MUMC+ hopes that other academic medical centers may benefit from this study and evaluation of the MOS.

## Author Contributions

EE drafted the original article based on interviews with AH and GM and materials provided by GM. Feedback and additions to the draft were provided by AH, WB and GM. AH and WB focused on the sections concerning the operating rooms and GM on the sections concerning the general organization of the hospital.

## Conflict of Interest Statement

The authors are employed by the Maastricht University Medical Center (MUMC+). The authors declare that the research was conducted in the absence of any other commercial or financial relationships that could be construed as a potential conflict of interest.

## Footnotes

^1^Mason et al. (6) describe Lean and Six Sigma as follows. Lean is a methodology “which uses an on-going cycle of improvement to focus on mapping out and adapting process pathways to preserve the steps that provide ‘value’ and to eliminate sources of waste.” Six Sigma meanwhile is a process “which aims to improve quality by identifying and correcting the causes of errors and in doing so reduce the rate to a six sigma level—3.4 defects per million opportunities (DPMO).” Combining these two results in Lean Six Sigma, which uses the steps described by the acronym DMAIC (Define, Measure, Analyze, Improve, Control) to benefit from both systems.

^2^The Toyota Production System has been developed by Toyota since the 1940s to guide the development and production of their products and services (10). It consists of many aspects, but can generally be described as “the relentless identification of defects in processes and the ongoing study of these defects while implementing trials toward their elimination” (11).
